# In Vivo Transcription Dynamics of the Galactose Operon: A Study on the Promoter Transition from *P1* to *P2* at Onset of Stationary Phase

**DOI:** 10.1371/journal.pone.0017646

**Published:** 2011-03-18

**Authors:** Sang Chun Ji, Xun Wang, Sang Hoon Yun, Heung Jin Jeon, Hee Jung Lee, Hackjin Kim, Heon M. Lim

**Affiliations:** 1 Department of Biological Science, College of Biological Sciences and Biotechnology, Chungnam National University, Taejon, Republic of Korea; 2 Department of Chemistry, College of Natural Sciences, Chungnam National University, Taejon, Republic of Korea; Ajou University, Republic of Korea

## Abstract

Quantitative analyses of the 5′ end of *gal* transcripts indicate that transcription from the galactose operon *P1* promoter is higher during cell division. When cells are no longer dividing, however, transcription is initiated more often from the *P2* promoter. *Escherichia coli* cells divide six times before the onset of the stationary phase when grown in LB containing 0.5% galactose at 37°C. Transcription from the two promoters increases, although at different rates, during early exponential phase (until the third cell division, OD_600_ 0.4), and then reaches a plateau. The steady-state transcription from *P1* continues in late exponential phase (the next three cell divisions, OD_600_ 3.0), after which transcription from this promoter decreases. However, steady-state transcription from *P2* continues 1 h longer into the stationary phase, before decreasing. This longer steady-state *P2* transcription constitutes the promoter transition from *P1* to *P2* at the onset of the stationary phase. The intracellular cAMP concentration dictates *P1* transcription dynamics; therefore, promoter transition may result from a lack of cAMP-CRP complex binding to the *gal* operon. The decay rate of *gal*-specific transcripts is constant through the six consecutive cell divisions that comprise the exponential growth phase, increases at the onset of the stationary phase, and is too low to be measured during the stationary phase. These data suggest that a regulatory mechanism coordinates the synthesis and decay of *gal* mRNAs to maintain the observed *gal* transcription. Our analysis indicates that the increase in *P1* transcription is the result of cAMP-CRP binding to increasing *numbers* of galactose operons in the cell population.

## Introduction

**Figure 1:**
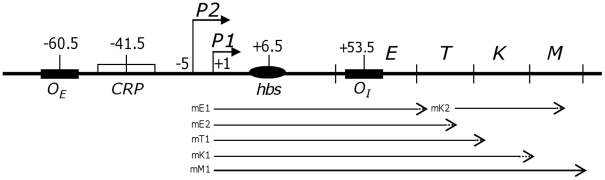
The galactose operon. Transcription initiation sites of the *P1* and *P2* promoters are indicated by arrows. The numbers indicate nucleotide position relative to the *P1* transcription initiation site (+1). The CRP-binding site is represented by an empty box labeled “CRP”. The two operator sites to which GalR binds are represented by black boxes labeled “*O_E_*” and “*O_I_*”. The black oval labeled “*hbs*” indicates an HU-binding site. The four structural genes of the *gal* operon are represented as E, T, K, and M, designating genes for epimerase (GalE), transferase (GalT), kinase (GalK), and mutarotase (GalM), respectively. The *gal* specific mRNA species are presented as arrows. Note that these mRNA species establish mRNA concentration gradient that is higher at the promoter-proximal region [9].

Genetic studies have demonstrated that the galactose operon of *Escherichia coli* has two promoters, *P1* and *P2*, which are separated by five nucleotides [1], [2]. These promoters are responsible for transcription of the four structural genes, *galE, galT, galK,* and *galM* (Fig. 1). Three trans-acting proteins, GalR (the *gal* repressor), cAMP receptor protein (CRP)-cAMP complex, and the histone-like protein HU, control transcription from these two promoters. Biochemical assays with purified components have shown that GalR binds to the two operator sequences, *O_E_* and *O_I_*, (Fig. 1) with equal affinity [3], [4]. In the presence of HU, GalR bound to *O_E_* and *O_I_* brings the two operators together to form a DNA loop, which simultaneously represses *P1* and *P2* [5]. When the *gal* operon is induced, the CRP-cAMP complex activates the *P1* promoter through direct contact between CRP and the N-terminal domain of the alpha subunit of the RNA polymerase (RNAP) holoenzyme [1], [6]. This contact increases the promoter-binding activity of RNA polymerase or facilitates open-complex formation, or may achieve both effects [7]. The CRP-cAMP complex appears to repress the *P2* promoter [1], [2].

Transcription of the *gal* operon produces the same gene products regardless of the promoter used. Despite the detailed understanding of this complex two-promoter system, the purpose of having two promoters for a single operon remains unclear. The *gal* operon must be transcribed under all growth conditions because Gal proteins are involved not only in galactose catabolism but also in the glycosylation of lipopolysaccharides in the outer membrane of *Escherichia coli* [8]. Thus, the two-promoter system may be needed to ensure continuous synthesis of Gal proteins under various external or internal conditions. In our previous study [9], we described an mRNA concentration gradient that is higher in the promoter proximal cistron than the distal region, and showed that transcription from the *P2* promoter generates a steeper mRNA concentration gradient than the *P1* promoter. We suggested that the steeper mRNA concentration gradient may account for the observation that a cAMP-deficient strain in which *P2* is known to be more active than *P1* [10] showed severe polarity in expression of the operon [11], [12], [13].

We reasoned that the amount and speed of transcription from the two promoters would be different. Thus, we evaluated *P1* and *P2* promoter usage in vivo and found that *P1* produces 70% of the total *gal* transcripts during exponential growth phase. However, during stationary phase, the promoter usage is reversed; 70% of the *gal* transcripts are produced from the *P2* promoter. To understand the observed promoter transition from *P1* to *P2* at the beginning of the stationary phase in molecular terms, we measured synthesis and decay rates of transcripts from the *P1* and *P2* promoters, and found that *P1* transcription was down regulated earlier than *P2* transcription at the end of the exponential growth phase.

## Results

### Promoter usage differs in growth and stationary phases

**Figure 2:**
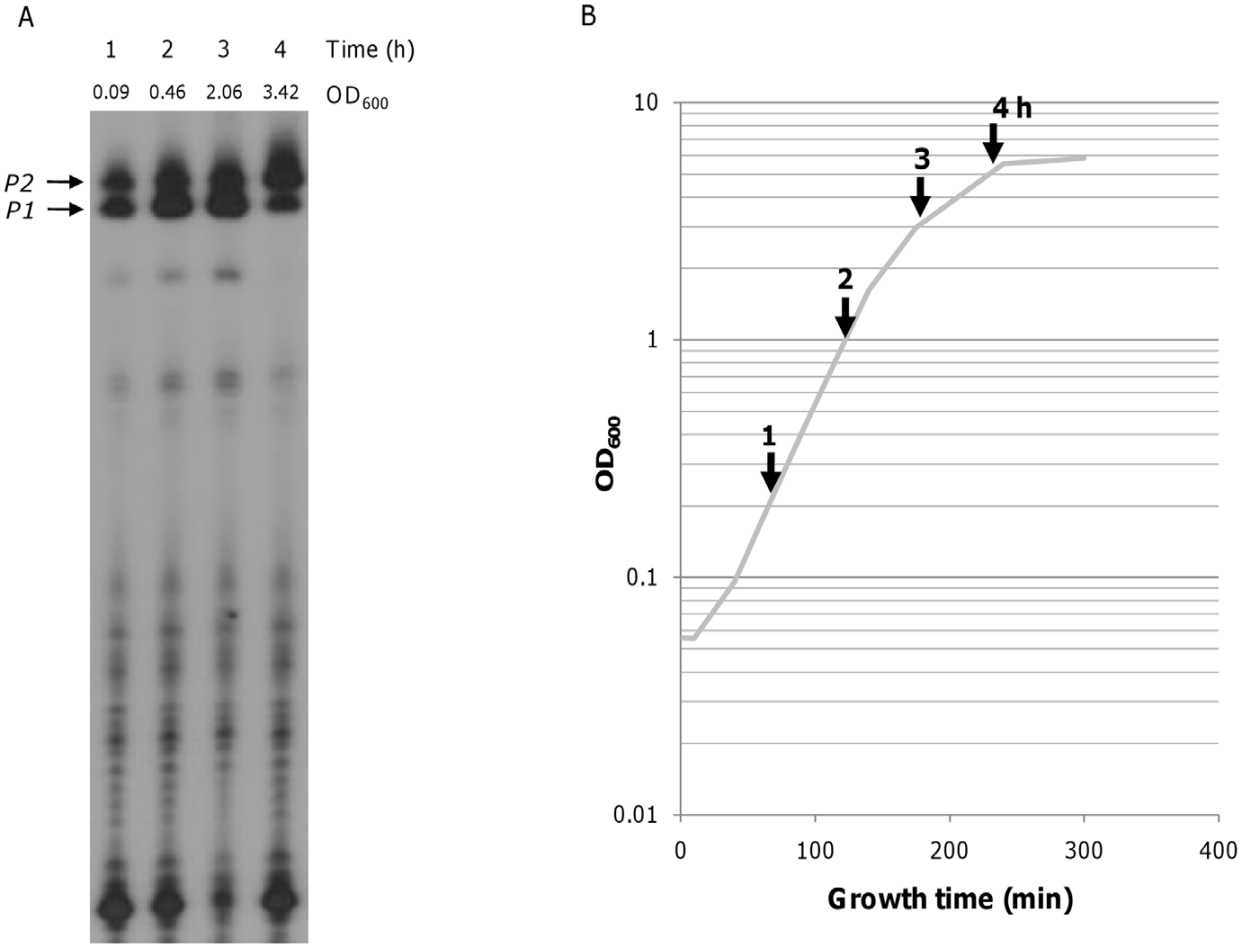
The ratio of *P1*-initiated and *P2*-initiated transcripts during different growth phases. The *gal* transcripts were analyzed by 5′-RACE and primer extension followed by 8% DNA sequencing gel electrophoresis (A). MG1655 (WT strain) was grown in LB containing 0.5% galactose. Total RNA was isolated from 1×10^8^ cells at different time points, indicated by vertical arrows in the growth curve (B) and analyzed by 5′-RACE. Each lane in (A) corresponds to the time point indicated in (B).

The relative number of transcripts initiated at the galactose operon *P1* and *P2* promoters were determined during different growth phases. To distinguish transcripts originating from *P1* and *P2*, which differ by only five bases at the 5′ end, we employed a modified 5′-RACE assay [14]. Briefly, total RNA was prepared from a fixed number of cells, usually 1×10^8^ cells (as determined by optical density and colony forming unit measurement). The 3′ hydroxyl ends of 5S rRNAs were ligated to the 5′-phosphate ends of mRNAs before reverse transcription, which enabled amplification of *P1-*specific and *P2*-specific cDNAs. After primer extension of a P^32^-labeled DNA primer, the 5′-ends of the *P1*-specific and *P2*-specific transcripts were visualized by electrophoresis on a DNA sequencing gel (Fig. 2A). These assays were performed using total RNA isolated from *E. coli* MG1655 (WT) grown in LB medium containing 0.5% galactose. Thus, we measured *P1*-initiated and *P2*- initiated transcripts in a fixed number of cells at different time points under *gal*-inducing conditions. Quantitative analysis of the *P1*-initated and *P2*- initiated transcripts with a PhosphorImager™ indicated that 70% of *gal*-specific transcripts were initiated from the *P1* promoter during the exponential growth phase, and 30% were initiated from the *P2* promoter (Fig. 2A, lanes 1–3). At the onset of stationary phase, however, 70% of the transcripts were generated from the *P2* promoter and 30% from the *P1* promoter (Fig. 2A, lane 4). This transition from *P1* to *P2* as the major promoter occurred at the transition to stationary phase (Fig. 2B).

### Cause of promoter usage transition from P1 to P2

To explore the mechanism(s) that causes promoter transition at onset of stationary phase, we performed the same experiments in two mutant strains: CH1106, which lacks the stationary phase-specific RNAP sigma factor RpoS [15], [16], and CF10237, which is deficient in guanosine tetraphosphate (ppGpp), a small molecule that redirects transcription to genes for starvation and survival [17], [18]. Promoter transition in the two mutant strains was identical to that of the WT strain (data not shown), suggesting that promoter transition is not due to preferential recognition of the *P2* promoter by the RNAP holoenzyme containing RpoS or by the increasing ppGpp concentration in the stationary phase.

**Figure 3:**
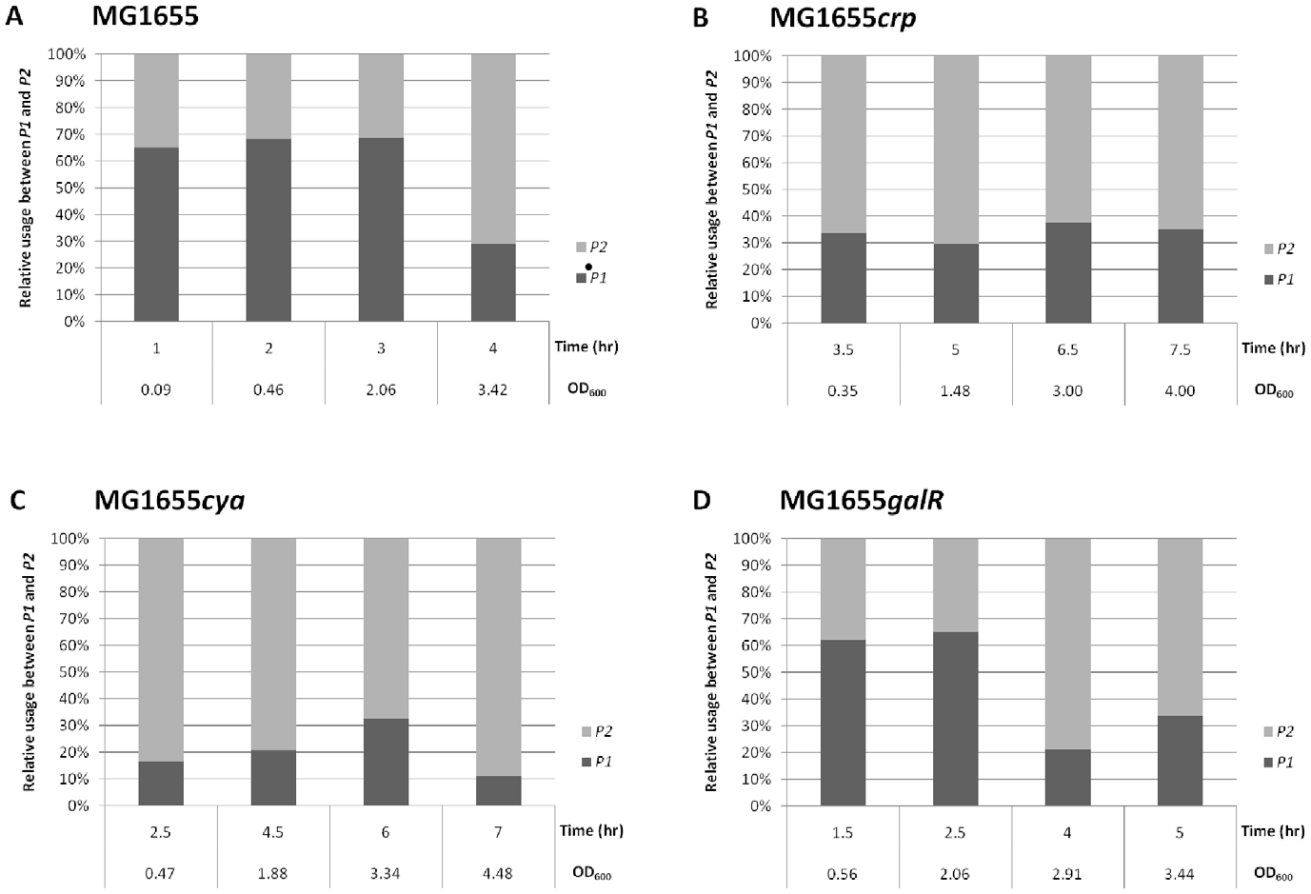
Effect of CRP, cAMP, and GalR on *gal* operon transcription dynamics. The ratio of *P1*-initiated and *P2*-initiated transcripts during different growth phases was evaluated in (A) MG1655 (WT), (B) MG1655*crp* (CRP-deficient), (C) MG1655*cya* (cAMP-deficient), and (D) MG1655*galR* (GalR-deficient) strains. The *gal* transcripts were analyzed by 5′-RACE and primer extension assay followed by 8% DNA sequencing gel electrophoresis. Amount of the transcripts from *P1* and *P2* promoters is shown in percent of the total *gal* transcripts.

We then performed 5′-RACE experiments using total RNA isolated from mutant strains that lack the CRP-cAMP and GalR transcription factors (Fig. 3). The CRP-deficient and cAMP-deficient strains both used *P2* as the major promoter throughout the growth phases, instead of switching from *P1* to *P2* at the transition to stationary phase (Fig. 3A and 3B). These data suggest that the CRP-cAMP complex is required for *P1* transcription in the exponential growth phase [2] and may be involved in promoter transition. In contrast, promoter transition of the *galR*-deficient strain (Fig. 3C) was similar to that of the WT strain, suggesting that GalR is not involved in promoter transition.

### Real-time RT-PCR analysis of the P1 and P2 promoter transcription dynamics

To compare the relative number of transcripts of samples taken at different time points, real-time RT-PCR is a better method than 5′-RACE because 5′-RACE is based on end-point PCR reactions. The range of cDNA concentrations that correspond to the linear range of real-time PCR amplification is 4 to 5 orders of magnitude higher than that of end-point PCR [19]. Thus, we used real-time RT-PCR to make more sensitive measurements of changes in *gal* transcription during different growth stages.

**Figure 4:**
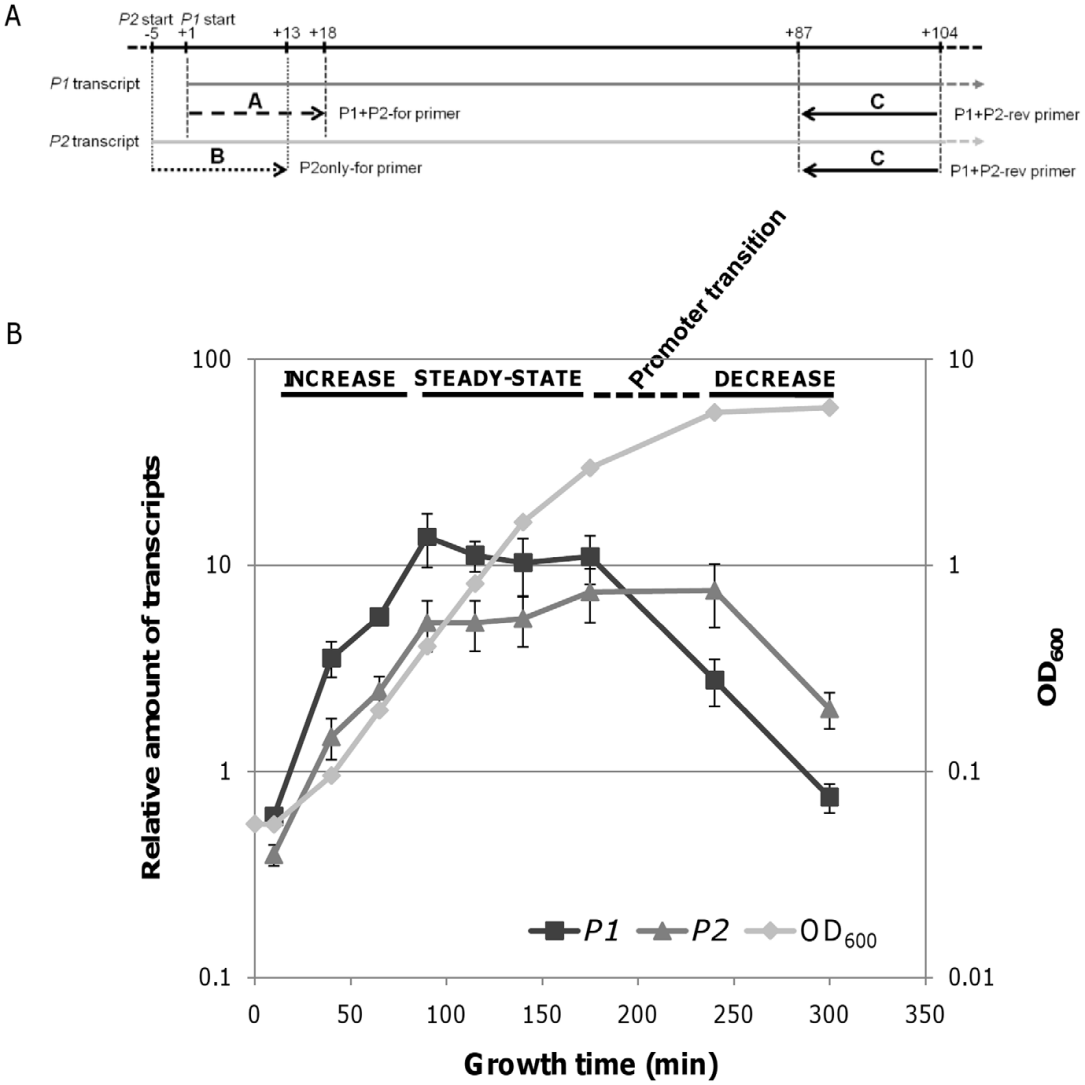
Analysis of *P1*-specific and *P2*-specific transcription dynamics during different growth phases. The relative number of *P1* and *P2* transcripts were determined by real-time RT-PCR using the DNA primers shown in (A) in the wild type strain MG1655. The primers anneal to the 5′ end of the *gal* operon and the numbers indicate nucleotide position relative to the transcription initiation site (+1) of the *P1* promoter. Thus, the resulting PCR products are about 100 bp long. (B) For real-time RT-PCR analysis, total RNA was prepared from equal numbers of cells (1×10^8^) for each time point (rectangles). Transcript levels are expressed relative to the first time point of wild type *P1* transcription. The error bars indicate standard deviation from three independent experiments (B).

To distinguish between transcripts that differed by only five bases in their 5′ ends by real-time RT-PCR, we used one reverse primer that annealed to the 3′ ends of both *P1* and *P2* transcripts, in combination with one of two forward primers: P2only-for, which annealed only to the *P2* transcript, and P1-P2-for, which annealed to both transcripts (Fig. 4A) [20]. The relative number of *P1*-initiated transcripts was calculated by subtracting the number of *P2* transcripts from the total number of transcripts.

### Promoter transition is due to differential transcription dynamics of P1 and P2 transcription

The relative number of *P1* and *P2* transcripts was plotted at time points during the exponential and stationary growth phases (Fig. 4B), revealing that *gal* transcription consists of three distinct periods we named INCREASE, STEADY-STATE, and DECREASE. The INCREASE and STEADY-STATE periods comprise the entire exponential growth phase. Transcription from both *P1* and *P2* promoters increases exponentially during the INCREASE period, which begins with the start of incubation (OD_600_ 0.05) and ends 90 min after the start of incubation (OD_600_ 0.4). Although the STEADY-STATE period of *P1* and *P2* transcription both begin at 90 min, STEADY-STATE *P1* transcription ends at 175 min (OD_600_ 3.0), which is the onset of the stationary phase. In contrast, STEADY-STATE *P2* transcription extends into the stationary phase, ending at 240 min (OD_600_ 4.6). The STEADY-STATE periods of *P1* and *P2* transcription lasted 85 and 150 min, respectively. Transcription decreases during the DECREASE period, which starts at 175 min for the *P1* transcription. Due to the longer STEADY-STATE period of *P2* transcription, however, the DECREASE period starts at 240 min, when cells are in early stationary phase.

The ratio of the relative number of *P1* and *P2* transcripts is 70/30 (*P1* transcript 70%, *P2* transcript 30%) throughout the entire exponential growth phase, as determined by real-time RT-PCR, but is reversed to 30/70 during the stationary growth phase (Fig. 4B). This result is consistent with results of the 5′-RACE assay, which suggested a promoter transition from *P1* to *P2* at the onset of the stationary growth phase (Fig. 2A). The real-time RT-PCR findings indicate that the longer STEADY-STATE period of *P2* transcription compared with that of *P1* transcription constitutes the promoter transition.

### Kinetic analysis of gal transcription during the INCREASE and STEADY-STATE periods

The exponential increase in transcription during the INCREASE period (Fig. 4B) indicated first-order kinetics. The first-order rate equation fits the data obtained from the first four time points (10, 40, 60, and 90 min) (R^2^>0.95). The rate constants for *P1* and *P2* transcription were 0.0372±0.0025 min^−1^ and 0.0312±0.0028 min^−1^, respectively, indicating that the number *P1* transcripts increased faster than the number of *P2* transcripts, resulting in the number of *P1* transcripts doubling every 18.7±1.2 min and the number of *P2* transcripts doubling every 22.3±1.9 min.

Since accumulation rate (observed rate)  =  synthesis rate – degradation rate, we measured the decay rate (degradation rate) of the *gal* transcripts to determine the actual transcription rate (synthesis rate). The rapid degradation of *E. coli* mRNAs [21] required measurement of *gal* mRNA decay rates during the different growth phases. We measured the decay rate of *P1* and *P2* transcripts separately. MG1655 cells were cultured in LB with 0.5% galactose, and rifampicin was added to stop transcription at various time points. The cells were analyzed 0, 2, 4, 6, and 8 min after adding rifampicin. To determine the decay rate and mRNA half-life, the relative number of *P1* and *P2* transcripts in each sample were determined by real-time RT-PCR.

**Figure 5:**
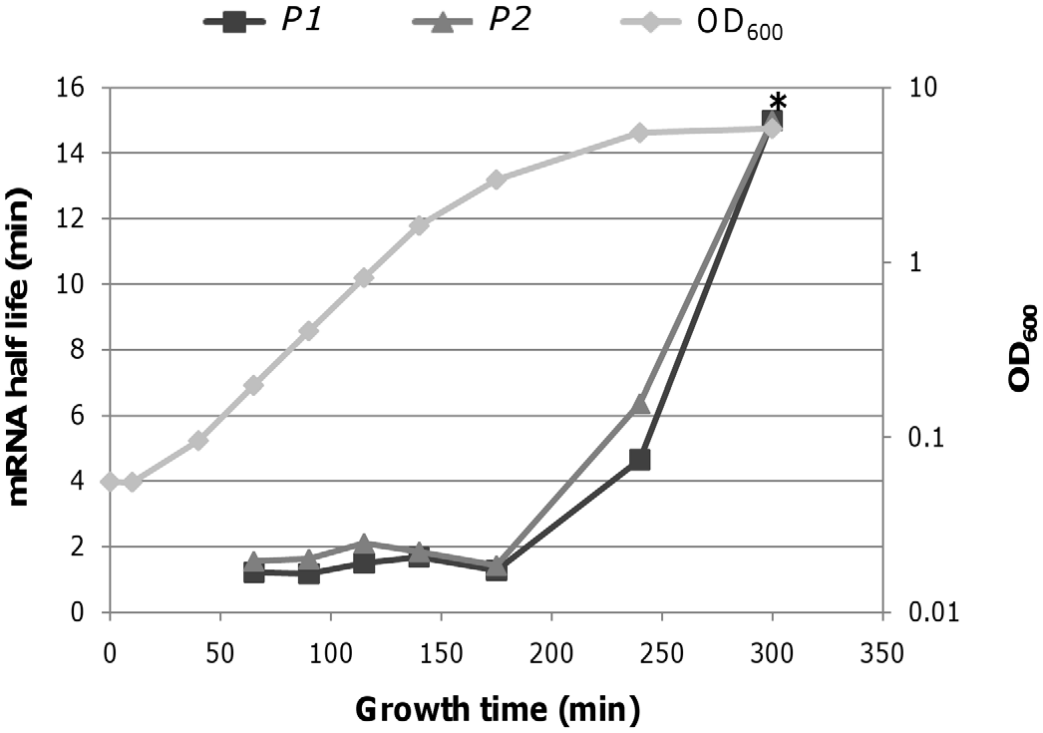
Half-life of the *gal* transcripts during different growth phases. Half-life of mRNA at each time point was determined by real-time RT-PCR. *decay rate was too low measure. The standard deviation from three independent experiments at time point of 65 min was too small to be represented in the scale of the figure.

As shown in Fig. 5, the half-lives of *P1* and *P2* transcripts were constant from 65 min to 175 min (OD_600_ 3.0): 1.37±0.22 min for *P1-*initiated transcription and 1.71±0.27 min for *P2-*initiated transcription. Therefore, the difference in the relative number of transcripts in the INCREASE and STEADY-STATE periods (Fig. 4B) are therefore due to changes in *P1-* and *P2*-transcription rate. Note that cells are still dividing at the same rate when the *gal* transcription rates are being changed.

Analyses based on decay rates suggest that actual transcription in vivo doubles every 1.28±0.01 min from the *P1* promoter and every 1.60±0.01 min from the *P2* promoter during the INCREASE period. The observed number of *P1* and *P2* transcripts (Fig. 4B) doubles every 18.7 and 22.3 min, respectively, indicating that the actual transcription during the INCREASE period is 15 to 16 times faster than the observed increase in transcript number to compensate for the rapid mRNA degradation. In vivo transcription during the STEADY-STATE period occurs at the same rate as mRNA decay. Thus, during the STEADY-STATE period, the number of *P1* transcripts doubles every 1.37 min and the number of *P2* transcripts doubles every 1.71 min. This rate of transcription from the *P1* promoter continues to 175 min, but continues to 240 min from the *P2* promoter, indicating differential regulation of the *P1* and *P2* promoters.

### Transcription during the DECREASE period

**Figure 6:**
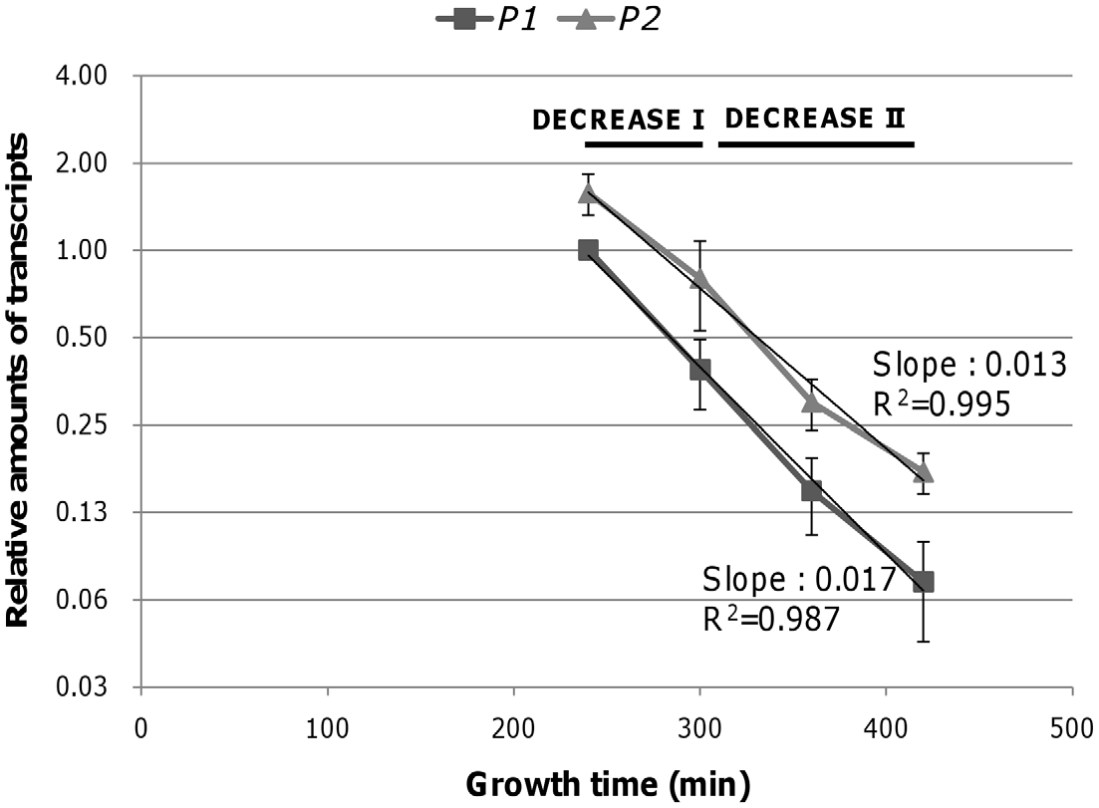
Transcription dynamics of *P1*-initiated and *P2*-initiated transcription during the DECREASE period. Relative numbers of *P1* and *P2* transcripts were determined at 240, 300, 360, and 420 min. Rate constants were determined from the slope, and fitted curves are presented. The error bars indicate standard deviation from two independent experiments

The linear decrease of transcription in the semi-log scale plot (Fig. 4B) during the DECREASE period indicated that the number *gal* transcripts decreased exponentially, suggesting first-order kinetics. To more accurately determine the decrease rate constant, we measured the relative number of *P1* and *P2* transcripts at 240, 300, 360, and 420 min (Fig. 6). The rate constant for *P1* was calculated as −0.017 min^−1^ and the rate constant for *P2* transcription as −0.013 min^−1^, indicating that the transcripts decrease by half every 40 and 53 min, respectively. At 240 min, the half-lives of the *P1* and *P2* transcript were 3.35 min, and 5.52 min, respectively, indicating that the decay was almost twice (*P1*) and three times (*P2*) slower than those of the exponential phase (Fig. 5). After 300 min, mRNA half-lives were too long to be measured; after rifampicin treatment, the number of mRNA transcripts changed very little, indicating that mRNA decay of the *gal* transcripts almost came to a halt after 300 min.

The DECREASE period was further divided into DECREASE I, in which the mRNA decay slows, and DECREASE II, in which there is virtually no mRNA decay. These mRNA decay dynamics showed that in the DECREASE I period, the number of transcripts initiated from *P1* doubled every 5.2 min (175–300 min) and the number of transcripts initiated from *P2* doubled every 7.1 min (240–300 min), demonstrating that transcription slowed down significantly compared with the STEADY-STATE period (Table 1). However, actual transcription slowed down even further during the DECREASE II period: reducing the number *P1* and *P2* transcripts by half required 40 min and 53 min, respectively (Table 1).

**Table 1:** Time required to double or reduce by half the number of *gal* transcripts during different growth phases.

Time (growth phase)	Transcription period	*P1* transcription rate (min)		*P2* transcription rate (min)	
		Observed rate^1^	Actual rate^2^	Observed rate	Actual rate
0–90 (early exponential)	INCREASE	18.7 (±1.2)	1.28 (±0.01)	22.3 (±1.9)	1.60 (± 0.01)
90–175 (late exponential)	STEADY-STATE	0	1.37 (±0.22)	0	1.71 (±0.22)
175–300 (early stationary)	DECREASE I	−40^a^	5.2	−53	7.1
>300 (stationary)	DECREASE II	−40	−40	−53	−53

1 Observed rate: rate of mRNA accumulation.

2 Actural rate: rate of actual transcription.

a negative sign (-) represents the time required to reduce the number of *gal* transcripts by half.

### Transcription from the P1 promoter is likely regulated by DNA binding of CRP-cAMP

**Figure 7:**
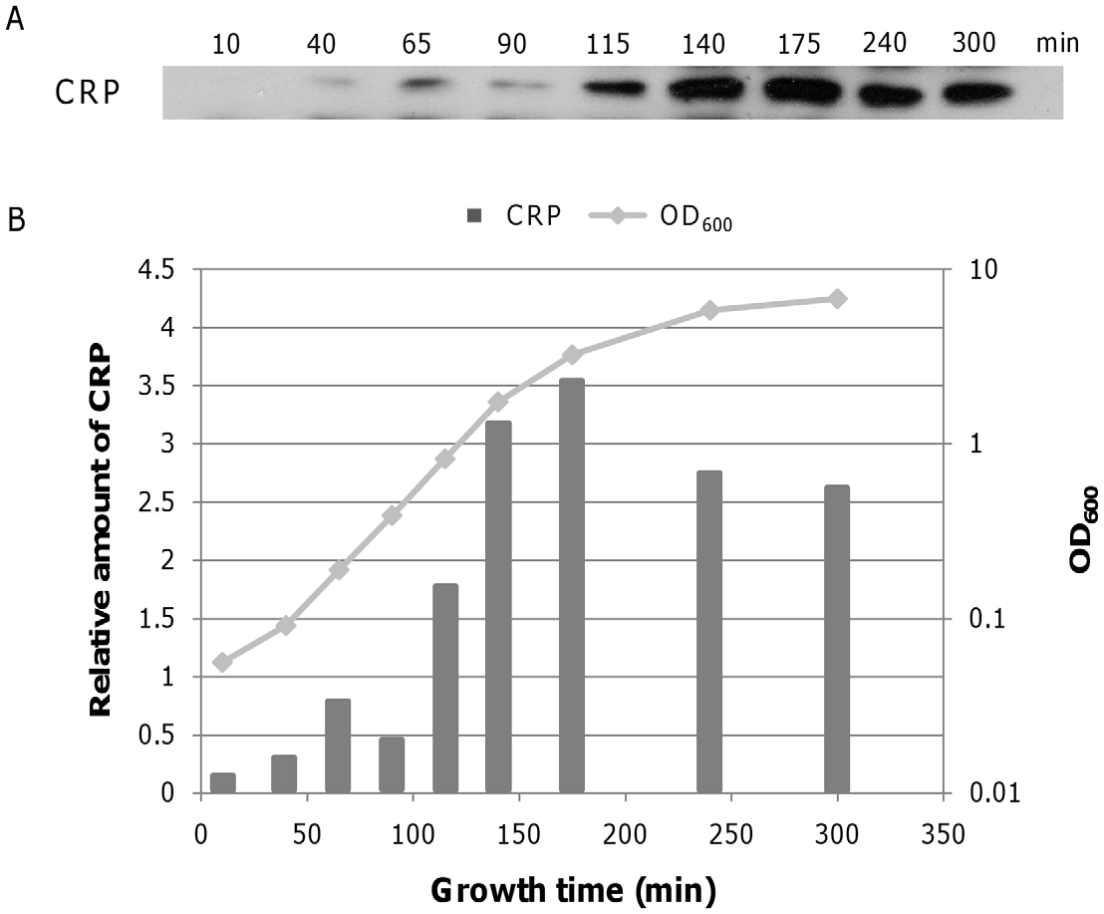
Dynamics of CRP concentration in different growth phases. Amount of CRP at different time points was determined by Western blot analysis (A), and plotted as a function of growth (B).

**Figure 8:**
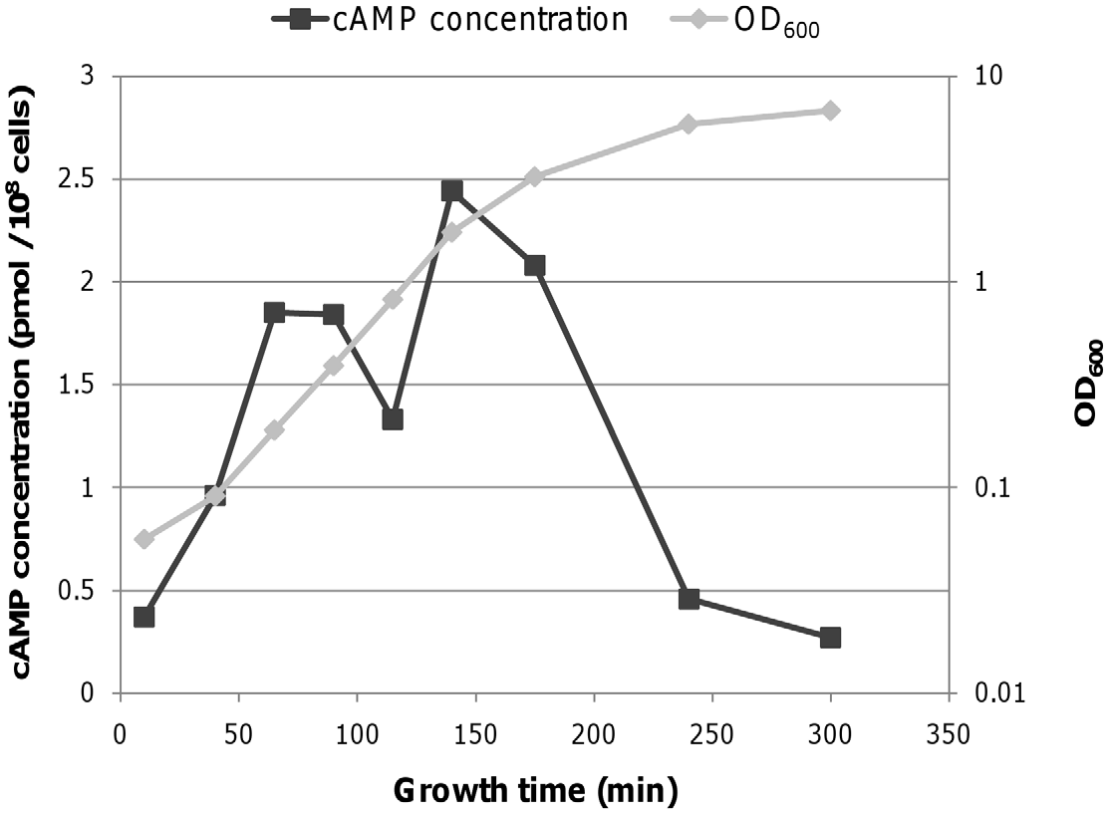
Dynamics of cAMP concentration at different growth phases. Intracellular cAMP concentrations at different time points were determined by an enzyme-linked immunoassay.

The *P2* transcription dominance during exponential growth in strains lacking CRP or cAMP (Fig. 3) suggested that the CRP-cAMP complex regulates *P1* transcription. To understand the role of the CRP-cAMP complex on *P1* transcription dynamics, we measured changes in the amount of CRP protein and cAMP during different growth phases in MG1655 (Figs. 7 and 8). As shown in Fig. 7, CRP concentration gradually increases with time, peaks at 175 min, and then decreases during the stationary phase. However, cAMP initially increases to four times its initial concentration, followed by a more or less steady-state period (Fig. 8). At 175 min, cAMP concentration starts to decrease, declining to 25% of peak levels by 240 min. The concentration dynamics of cAMP, rather than CRP, appear to be more closely related to *P1* transcription dynamics. The initial increase of both CRP and cAMP suggests that increased binding of the CRP-cAMP complex to the *gal* operon promoter region caused the initial increase in *P1* transcripts during the INCREASE period (Fig. 4B). Dissociation of the CRP-cAMP complex from the *gal* operon due to lower cAMP concentrations at the onset of the stationary phase may have decreased *P1* transcription, resulting in promoter transition.

### Rsd and 6S RNA do not affect gal transcription during the DECREASE period

The kinetic analysis of the *gal* transcription in vivo showed that transcription from both promoters is down regulated (Fig. 4B). We were interested if there is any transcription factors (other than CRP-cAMP) involved in down regulation of the *gal* transcription in stationary phase. Recently, 6S RNA was shown to specifically down-regulate promoters with a weak -35 element and an extended -10 element such as the *gal* promoters [22] during stationary phase. The *E. coli* protein Rsd [23] inhibits transcription initiated by sigma70 during the stationary phase [24], [25]. We determined the transcription dynamics of the *gal* operon in strains deficient in 6S RNA (*ssrS1*) or the Rsd protein (*rsd*) to assess whether these factors are involved the down-regulation of *gal* transcription during the DECREASE period. We expected to see little or no decrease in *gal* transcription during the DECREASE period if these factors affected *gal* transcription during the stationary phase. However, decreasing transcription rates from both promoters was similar to that of WT (Fig. 6, data not shown). We concluded that these factors are not involved in down-regulating *gal* transcription, at least not during the early stationary phase.

## Discussion

### Physiological consequences of the promoter transition

It has been known that the genes proximal to the promoter produce more proteins than the ones distal to the promoter, a phenomenon known as “polarity” in gene expression [26], [27], [28]. The fundamental cause of the polarity effect seems to reside on what has been defined as “mRNA concentration gradient”, in which the concentration of the promoter-proximal mRNA is greater than that of the promoter-distal mRNA [9]. The mRNA concentration gradient could be established because different species of the *gal* transcripts bear their 3′-ends at the end of each cistron of the *gal* operon (Fig. 1). Interestingly, the severity of mRNA gradient depends on which promoter the transcription initiates; transcription from *P1* generates steeper mRNA gradient than that from *P2* [9]. Thus, one of the reasons as to why *P2* becomes a dominant promoter during stationary phase might be that cells need more proteins from the front part of the operon, specifically GalE, UDP-galactose epimerase that catalyzes UDP-galactose to UDP-glucose. Indeed, the relationship between *P2* transcription and GalE production has been elegantly demonstrated in a recent report by Lee et al. [29]. The *P2* promoter is specifically derepressed (by low concentration of UTP, not by the conventional derepression mechanism by galactose) to produce more GalE protein when UDP-galactose concentration in the cell becomes high [29]. Another interesting previous result regarding *P2* transcription and GalE production is that the *P2* transcript can produce 3 times more of GalE protein than the *P1* transcript [13]. Thus, it is likely that one of the physiological consequences of the *P2* becoming the major promoter in *gal* transcription at the beginning of stationary phase is to maintain a certain level of GalE in a situation where the overall *gal* transcription decreases as shown in Fig. 4. Indeed, the amount of GalE measured by Western blot analysis stayed almost the same starting from late exponential to stationary phase while those of GalT and GalK decreased (unpublished result, HM Lim), supporting our notion that cells need to maintain a certain concentration of GalE against decreasing mRNA level during stationary phase.

### The actual transcription rates of the two promoters

The transcription dynamics shown in Fig. 4B represent changes in the relative number of *gal* transcripts from the *P1* and *P2* promoters during different growth phases. By measuring mRNA degradation, we were able to show the actual transcription rate required to account for these observed transcript levels. The actual transcription rates during different growth phases along with the observed changes in transcript levels are summarized in Table 1. The actual and observed transcription rates are expressed as time (min) required for the number of *gal* transcripts to double or be reduced by half. For example, the time required to double the number of transcripts initiated from *P1* was 1.28 min during the INCREASE period, 1.37 min during the STEADY-STATE period, and 5.2 min during the DECREASE I period. During the DECREASE II period, however, it required 40 min to reduce the amount of *P1* transcripts by half, and 53 min to reduce the number of *P2* transcripts by half. Because the decay rates of transcripts from both promoters were too low to be measured during this period (Fig. 5), it is likely that transcription from the both promoters ceased during the DECREASE II period.

The actual transcription rates from the two promoters show that transcription from *P1* is faster than transcription from *P2* throughout exponential growth. Our findings also suggested that the binding kinetics of the CRP-cAMP complex to the *gal* operon DNA may account for differences between transcription rates from the two promoters. Thus, the faster transcription of *P1* during the INCREASE period appears to be due to the activity of cAMP-CRP on the *P1* promoter during that time.

### Increased number of gal operons transcribed in the cell population

Because the mRNA decay rate remains constant throughout the exponential growth phase, we investigated the reason for the increased number of transcripts during the INCREASE period. This result could be achieved by increased RNAP initiation from a single *gal* operon over time. Alternatively, more *gal* operons within the cell population may be transcribed. The CRP-cAMP complex enhances transcription from the *P1* promoter by recruiting RNAP to a single *gal* operon [30] and promoting more rapid open complex formation [31]. At the same time, CRP-cAMP complexes may bind to more *gal* operons in the cell population. The increased levels of CRP protein and cAMP during the INCREASE period (Figs. 6 and 7) support both possibilities. Stochastic gene expression models [32] suggest that during early exponential growth (OD_600_ 0.05), few *gal* operons in the cell population are engaged in transcription.

The INCREASE period of *P1* transcription is followed by the STEADY-STATE period from 90 min (OD_600_ 0.4) to 175 min (OD_600_ 3.0), during which the transcript doubling time increases slightly from 1.28 min to 1.37 min, and there is no net increase in *P1* transcripts. We hypothesize that the number of *P1* transcripts reached a plateau because the number of activated *gal* operons did not change during this time. This hypothesis fails to explain why the transcription rate has changed during the STEADY-STATE period, but may explain the decrease in *P1* transcription during the DECREASE period.

Although CRP-cAMP appears to control *P1* transcription dynamics, the regulation of *P2* transcription is unclear. Results of in vitro experiments suggest GalR as a transcriptional activator for the *P2* promoter [33], but the binding dynamics of sigma70-RNAP to the *P2* promoter may also be involved.

### Models describing RNA polymerase on gal operon DNAs

**Figure 9:**
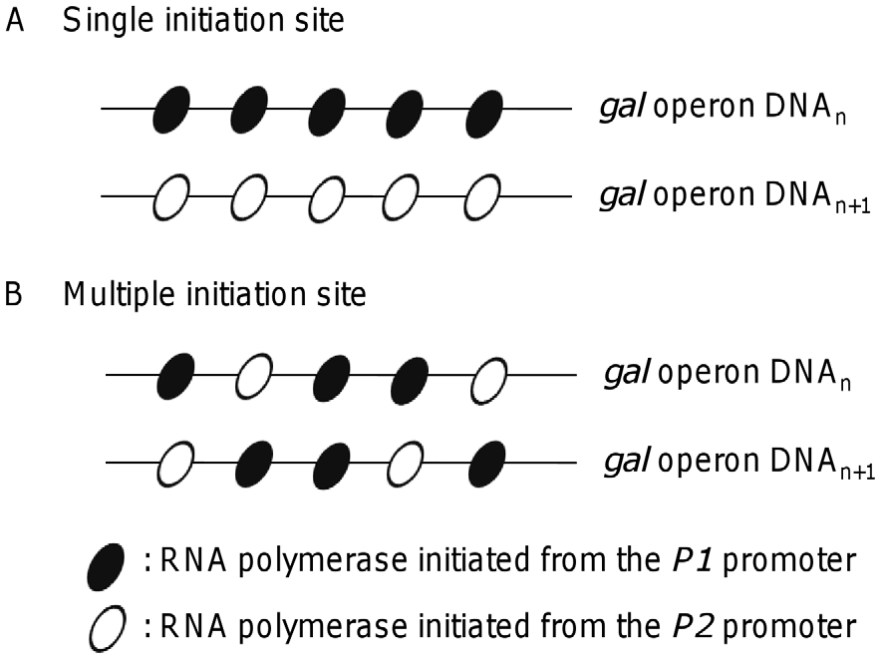
Models describing RNA polymerase on *gal* operon DNAs in the cell population. (A) Single initiation site model: each *gal* operon in the cell population is transcribed from the *P1* promoter only or from the *P2* promoter only. (B) Mixed initiation site model: each *gal* operon in the population is transcribed from both promoters, but at a different frequency from each promoter.

We demonstrated that under conditions that induce the *gal* operon, 70% of *gal* transcripts are initiated from the *P1* promoter in exponentially growing *E. coli* cells, and the remaining 30% are from the *P2* promoter. Two possible explanations exist for this distribution of transcripts. In the first model, 70% of the transcriptionally active *gal* operons in the population produce only *P1* transcripts (*P1*-initiating operons), and 30% produce only *P2* transcripts (*P2*-initiating operons). Thus, RNAPs transcribing a *gal* operon during a given period used either the *P1* or the *P2* transcription start site (Fig. 9A). An alternative possibility is that a *gal* operon produces 70% *P1* transcripts and 30% *P2* transcripts. In this second model, 70% of the RNAPs initiated transcription at *P1* and 30% of the RNAPs initiated transcription at *P2* in the same operon (Fig. 9B). The single initiation site model predicts that once a transcription factor binds to its DNA binding site, it remains bound for a long period of time, probably until an intracellular signal induces detachment [34]. In contrast, if *P1* and *P2* are both used on the same operon DNA, then transcription factors would be expected to transiently bind the DNA and fall off repeatedly. The single initiation model suggests that transcription from the two promoters is likely to occur in different *gal* operons in different cells or different *gal* operons within a single cell during exponential replication when multiple chromosome copies exist. We are currently testing these two models using the GFP gene cloned under the *gal* promoter control.

## Materials and Methods

### Bacterial strains

The bacterial strains used in this study were MG1655, MG1655*galR*, MG1655*crp*, MG1655*cya*, MG1655*rsd*, and MG1655*ssrS*. These mutant strains were generated by deleting the corresponding gene from MG1655 by λ red-mediated recombination [35]. The *rpoS* mutant strain CH1106 (GN122 *katF*::*Tn10*) and the ppGpp-deficient strain CF10237 (MG1655 *relA spoT*) were provided by H. E. Choy (Chonnam National University, Korea).

### Cell and RNA preparation

*E. coli* cells were grown in LB containing 0.5% galactose at 37°C with shaking (250 rpm). A fresh 100-ml culture was started by 1∶100 dilution of an overnight culture in LB with 0.5% galactose. To analyze the same number of cells (1×10^8^) at various time points during the exponential growth phase, the aliquot taken from the bacterial culture was halved at every doubling time (as assessed by optical density): at 10 min, 8 ml cells were taken; at 40 min, 4 ml cells were sampled, and so on until the end of the exponential growth phase (175 min). After 175 min, a volume corresponding to 10^8^ cfu was taken. Cells were harvested by centrifugation and resuspended in 50 µl protoplasting buffer (15 mM Tris-HCl [pH 8.0], 0.45 M sucrose, 8 mM EDTA). Lysozyme (5 µl, 50 mg/ml) was added, and the cells were incubated for 5 min at 25°C. A phenolic detergent (500 µl, TRI Reagent, Molecular Research Center) was added, and the sample was mixed by vortexing for 20 sec and then incubated for 5 min at 25°C. Samples were stored at -70°C overnight. The next day, the samples were thawed at room temperature, and RNA was purified from all samples simultaneously. Chloroform (100 µl, Sigma) was added to the samples, which were vortexed vigorously for 20 sec. After incubation for 10 min at 25°C, the samples were centrifuged at 12 000×*g* for 15 min at 4°C. The aqueous phase (250 µl) was then transferred to a new tube and mixed with 250 µl isopropanol (Sigma). After the samples were incubated for 10 min at 25°C, the RNA was collected by centrifugation at 12 000 × *g* for 8 min at 4°C and washed with 1 ml 75% cold ethanol. The precipitated RNA was dissolved in 50 µl of RNA storage buffer (Ambion). RNA concentration was determined by measuring absorbance at 260 nm using the NanoDrop™ spectrophotometer.

### Rapid amplification of cDNA ends of gal mRNA

Rapid amplification of cDNA ends (5′-RACE) was initiated by treating total RNA with tobacco acid pyrophosphatase (Epicentre, USA). To ligate the 3′-hydroxyl end of 5S rRNA to the 5′ phosphate end of *gal* mRNA, a 25-µl reaction containing 10 µl total RNA, 2.5 µl 10× reaction buffer (Ambion), 10 units T4 RNA ligase (Ambion), and 20 units RNasin (Promega) was incubated at 37°C for 4 h. This RNA was purified with a G-50 column (Amersham Biosciences). Using this RNA preparation, real-time reverse transcription-polymerase chain reaction (RT-PCR) and primer extension were performed as previously described [20]. The 5′ RACE assay was used to distinguish mRNAs transcribed from *P1* (70-bp) from those transcribed from *P2* (75-bp), which differs by five bases at the 5′ end.

### Real-time RT-PCR and quantification of P1 and P2 transcripts

Genomic DNA was removed from the RNA samples by Turbo DNA-free™ (Ambion) according to the manufacturer's protocol. The cDNA template was synthesized in a 20-µl reaction containing 2 µg DNase-treated RNA, 4 µl 5X reaction buffer (Toyobo, Japan), 1 µl primer mix, and 1 µl enzyme mix containing ReverTra Ace® reverse transcriptase and RNase inhibitor. After incubating at 37°C for 1 h, the reaction was stopped by heating at 98°C. Primers were designed by Primer 3 software [36]: P1+P2-for, 5′-ATA CCA TAA GCC TAA TGG-3′; P2only-for, 5′-ATT TCA TAC CAT AAG CCT-3′; P1+P2-rev, 5′-ATC ATG ACC GTT TTG CAG-3′. PCR conditions were optimized and all PCR primer sets had same amplification efficiencies. The 10-µl PCR reactions contained 5 µl iQ™ SYBR® Green Supermix (Bio-Rad), 3.6 µl nuclease-free water, 0.2 µl each forward primer (10 µM) and reverse primer (10 µM), and 1 µl cDNA template. Amplification was carried in a CFX96™ system (Bio-Rad) under the following conditions: initial denaturation at 95°C for 3 min, and 35 cycles of denaturation for 10 sec of at 95°C, annealing for 20 sec of at 55.5°C, and elongation for 15 sec at 72°C.

### Measurement of mRNA decay

To measure the decay rate of *gal* transcripts at different growth phases, MG1655 cells were grown as described above. At each sampling time, rifampicin was added to stop the initiation of transcription (final concentration, 500 µg/ml), and cells were analyzed 0, 2, 4, 6, and 8 min after rifampicin treatment. Harvested cells (10^8^ at each time point) were mixed immediately with 10% buffer-saturated phenol in ethanol (1/10 volume) and chilled rapidly to 4°C [21]. RNA preparation, cDNA synthesis, and real-time PCR were performed as described above. The amount of mRNA was plotted against time to determine the decay rate.

*Western blot analysis-* The CRP antibody was kindly provided by H. Aiba (Nagoya University, Japan). For Western blots, harvested cells (1×10^9^ cells) were resuspended in 300 µl SDS gel-loading buffer (50 mM Tris-Cl [pH 6.8], 100 mM dithiothreitol, 2% (w/v) SDS, 0.1% (w/v) bromophenol blue, 10% (v/v) glycerol, 8 mM MgCl_2_) and placed in a boiling-water bath for 3 min. The resulting crude cell lysate (10 µl) was separated by 10% polyacrylamide gel electrophoresis. Western blotting was performed as described in the standard cloning manual [37] using the ECL kit (Amersham Biosciences). The film was analyzed with the Gel Doc imaging system (Bio-Rad).

### Determination of intracellular cAMP concentration

To determine intracellular cAMP concentrations at different time points, cells were harvested by centrifugation at 12 000×*g* for 1 min at 4°C. The cells were then resuspended in 20 µl distilled water and boiled for 5 min. The samples were centrifuged at 12 000×*g* for 5 min at 4°C, and the supernatant (20 µl) was transferred to a new tube and mixed with 60 µl ethanol (Merck, Germany). The mixture was stored at −20°C. Before analysis, the mixture was dried completely by a centrifugal concentrator and resuspended in 200 µl cAMP assay buffer (GE Healthcare, USA). Determination of cAMP concentration was performed with the cAMP Biotrak enzyme immunoassay system (GE Healthcare).

### Transcription Kinetics

Kinetics of *P1* and *P2* transcription showed linear time dependence in the semi-log plot, indicating first-order kinetics. Rate constants were determined from the slope of the semi-log plot, and half-lives were calculated from the rate constants. The change in the number of transcripts (R) over time was:
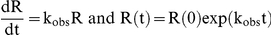
where k_obs_ is the observed first-order rate constant. However, the observed rate is not the actual increase in transcription because the first-order decay of transcripts during this period was not taken into account. The actual rate constants are given by:
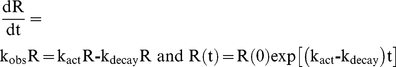
where k_act_ is the actual transcription rate constant and k_decay_ is the actual decay rate constant. The transcript half-life is related to the rate constant as τ = (ln2)/k. The actual doubling time during the INCREASE period was calculated as 1/τ_act_ = 1/τ_obs_ + 1/τ_decay_. The actual doubling time and half-life during the STEADY-STATE and DECREASE period were calculated in the same way.
